# Supplementary material for: Evidence for Quasi-High-LET Biological Effects in Clinical Proton Beams That Suppress c-NHEJ and Enhance HR and Alt-EJ
Source: Cells. 2026 Jan 4;15(1):86. doi: 10.3390/cells15010086 (PMC12785619; doi:10.3390/cells15010086)
Supplement: Supplementary file 1 [file cells-15-00086-s001.zip › cells-4053052-supplementary.pdf]

# SUPPLEMENTARY INFORMATION

## Supplementary figure legends

**Figure S1. (A)** Replotted clonogenic survival analysis of DSB repair proficient AA8 cells. **(B)** Same as in panel A, but for XR-C1-3 cells deficient for c-NHEJ. **(C)** Same as in panel A, but for Irs1SF cells deficient for HR. Data is obtained and replotted from reference [27].

**Figure S2. (A)** Representative indirect immunofluorescent (IF) images of A549 cells, exposed to increased doses of X-rays or SOBP protons. The γH2AX foci channel is rendered in red, while RAD51 foci are depicted in magenta. The EdU stained cell (green), indicate the cells in S-phase at the time of radiation, while the DAPI (blue), counterstained the nucleus. **(B)** Images captured from the IF experiment are processed by the image segmentation software to determine the number of γH2AX and RAD51 foci, together with total intensities of the EdU and DAPI channels. This information is fed to the flow cytometry software, that allows to gate the cells of interests. The dot plots, depicts the gates applied to select for $EdU^-$, $G_2$-phase cells. **(C)** Dot plots showing the distribution of the γH2AX foci within the selected $EdU^-$, $G_2$-phase cells population, determined by the gates applied.

**Figure S3.** Representative flow cytometry histograms, showing the distribution of the A549 cells, collected at the indicated time intervals, and processed for H3-pS10 and PI staining after irradiation with 1 Gy SOBP protons. Lower panels indicate the gates applied to differentiate the H3-pS10 positive cells and calculate the MI. The cells with $G_2$-phase DNA content are selected.

## Supplementary table legend

**Table S1.** Numerical values used to plot the clonogenic survival of DSB repair proficient (AA8) and DSB repair deficient (XR-C1-3 and Irs1SF) hamster cell lines. The tables also include the values for the RBA calculated for X-rays (X) and entrance protons (E) as well as for X-rays (X) and SOBP protons (SOBP). The RBE is calculated at 50%, (D50), 37% (D37), 10% (D10), and 1% (D1) survival.

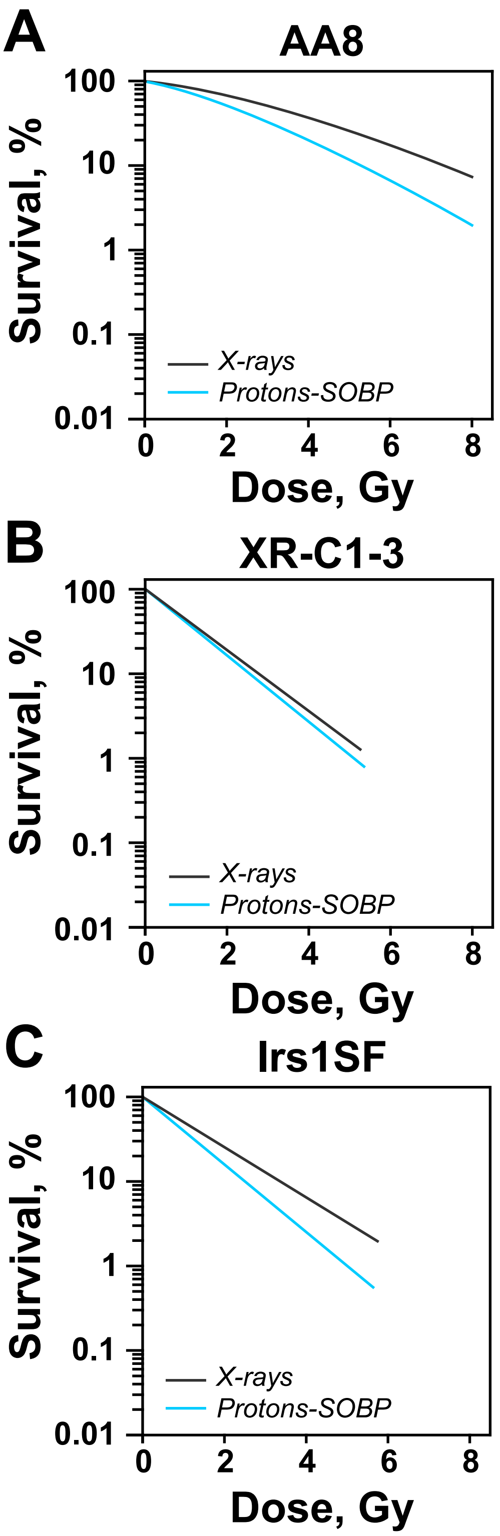


**Mladenov et al. Figure S1**

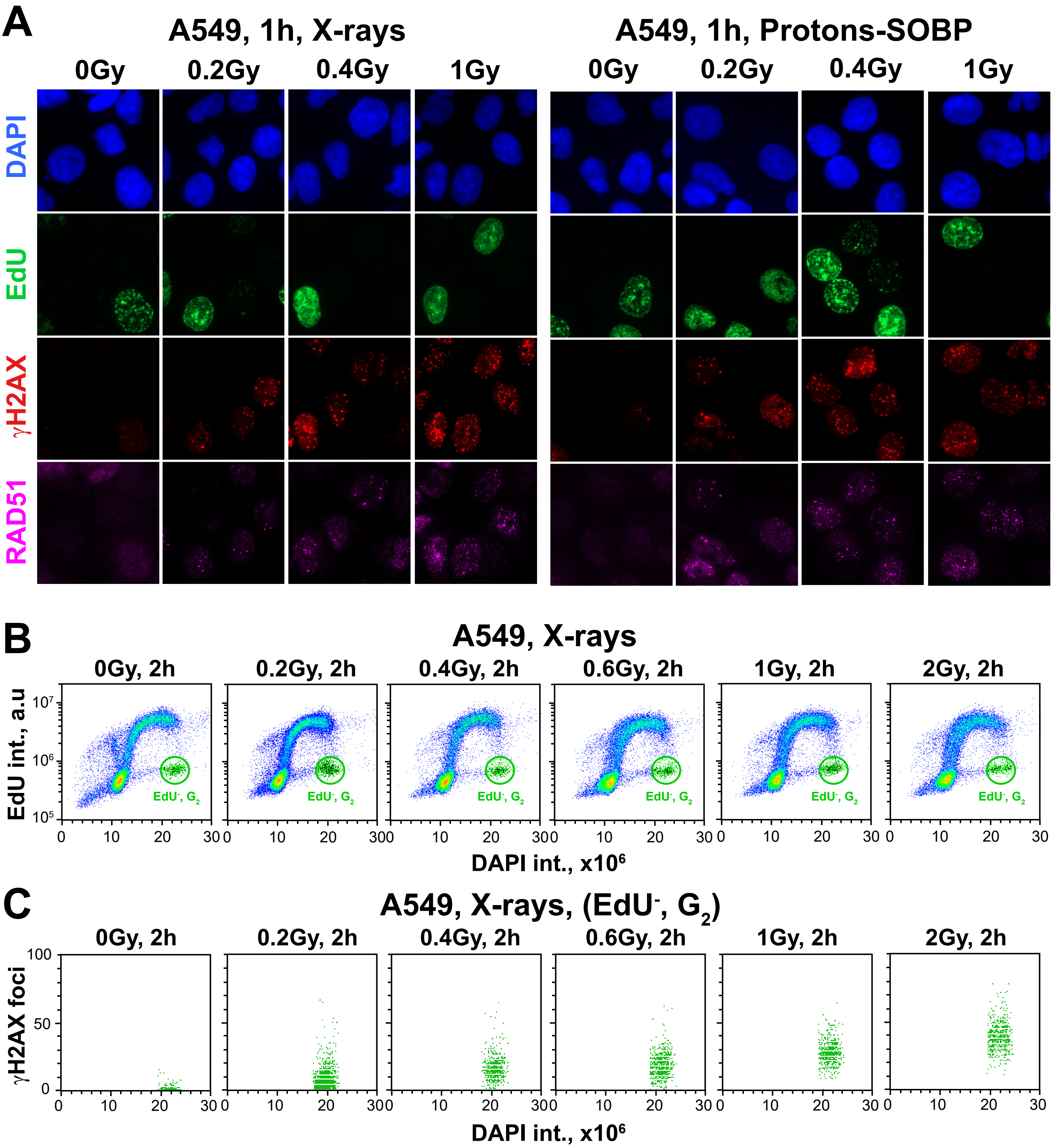


Mladenov et al. Figure S2

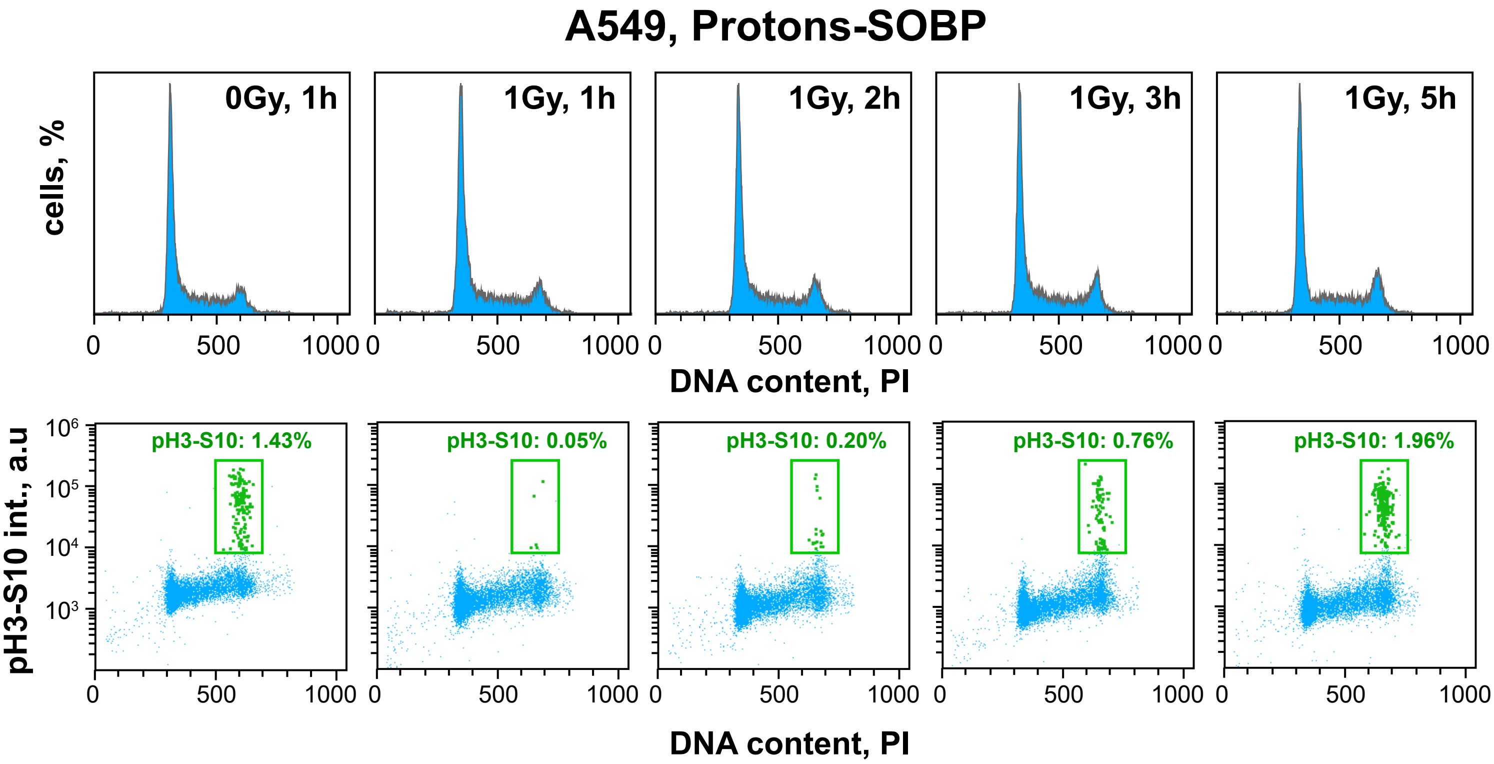


Mladenov et al. Figure S3

## AA8

| X-rays | | protons-E | | protons-SOBP | |
|---|---|---|---|---|---|
| SF, % | StDev | SF, % | StDev | SF, % | StDev |
| 100 | 0 | 100 | 0 | 100 | 0 |
| 83.72486 | 6.695855 | 81.38527 | 7.48349 | 82.94714 | 2.576697 |
| 59.09555 | 12.82517 | 63.75316 | 5.370886 | 53.3056 | 7.439763 |
| 27.10728 | 3.242062 | 37.87701 | 4.436904 | 30.50941 | 7.987012 |
| 6.338121 | 1.032693 | 11.55964 | 1.792157 | 6.64326 | 1.655808 |
| 1.378249 | 0.380155 | 1.943879 | 0.335768 | 1.282192 | 0.449709 |

| RBE (X vs E) | | RBE (X vs SOBP) | |
|---|---|---|---|
| D50 | 0.719295 | D50 | 0.95369 |
| D37 | 0.750099 | D37 | 0.960986 |
| D10 | 0.846088 | D10 | 0.985777 |
| D1 | 0.945513 | D1 | 1.013876 |

## XR-C1-3

| X-rays | | protons-E | | protons-SOBP | |
|---|---|---|---|---|---|
| SF, % | StDev | SF, % | StDev | SF, % | StDev |
| 100 | 0 | 100 | 0 | 100 | 0 |
| 43.33869 | 7.020354 | 44.27305 | 6.861033 | 48.65758 | 9.228299 |
| 15.62467 | 3.449973 | 17.86044 | 1.715252 | 17.08819 | 1.919308 |
| 6.356841 | 2.687422 | 7.562256 | 1.463828 | 5.20975 | 1.368936 |
| 0.659373 | 0.300751 | 0.666703 | 0.155338 | 0.586782 | 0.26352 |
| 0.037285 | 0.005438 | 0.041688 | 0.012959 | 0.0684 | 0.036558 |

| RBE (X vs E) | | RBE (X vs SOBP) | |
|---|---|---|---|
| D50 | 0.953471 | D50 | 1.100295 |
| D37 | 0.954729 | D37 | 1.094989 |
| D10 | 0.960456 | D10 | 1.069905 |
| D1 | 0.971721 | D1 | 1.015056 |

## Irs1SF

| X-rays | | protons-E | | protons-SOBP | |
|---|---|---|---|---|---|
| SF, % | StDev | SF, % | StDev | SF, % | StDev |
| 100 | 0 | 100 | 0 | 100 | 0 |
| 47.83354 | 6.801092 | 42.406 | 13.72564 | 44.82054 | 16.11537 |
| 25.17155 | 1.734609 | 16.30639 | 7.868137 | 20.35742 | 9.622363 |
| 9.515591 | 1.212767 | 5.793291 | 3.273789 | 4.828216 | 2.524957 |
| 0.80223 | 0.246914 | 0.188822 | 0.057609 | 0.17122 | 0.081621 |
| 0.104105 | 0.038658 | 0.027197 | 0.002867 | 0.023369 | 0.005255 |

| RBE (X vs E) | | RBE (X vs SOBP) | |
|---|---|---|---|
| D50 | 1.32704 | D50 | 1.334233 |
| D37 | 1.325204 | D37 | 1.332958 |
| D10 | 1.316493 | D10 | 1.326924 |
| D1 | 1.297385 | D1 | 1.31379 |

**Mladenov et al. Table S1**
